# Bridging the Gut Microbiota and the Brain, Kidney, and Cardiovascular Health: The Role of Probiotics

**DOI:** 10.1007/s12602-025-10680-6

**Published:** 2025-09-06

**Authors:** Milena Rosa Lopes, Rosa Direito, Elen Landgraf Guiguer, Vitor Cavallari Strozze Catharin, Tereza Lais Menegucci Zutin, Claudio José Rubira, Virgínia Maria Cavallari Strozze Catharin, Kátia Portero Sloan, Lance Alan Sloan, José Luiz Yanaguizawa Junior, Lucas Fornari Laurindo, Sandra Maria Barbalho, Ricardo de Alvares Goulart

**Affiliations:** 1 Department of Biochemistry and Pharmacology, School of Medicine, Universidade de Marília (UNIMAR), Marília, SP 17525-902 Brazil; 2 Laboratory of Systems Integration Pharmacology, Clinical and Regulatory Science, Research Institute for Medicines (iMed.ULisboa), Universidade de Lisboa, 1649-003 Lisbon, Portugal; 3 Postgraduate Program in Structural and Functional Interactions in Rehabilitation, School of Medicine, Universidade de Marília (UNIMAR), Marília, SP 17525-902 Brazil; 4 Department of Biochemistry and Nutrition, School of Food and Technology of Marília (FATEC), Marília, SP 17506-000 Brazil; 5 Laboratory for Systematic Investigations of Diseases, Department of Biochemistry and Pharmacology, School of Medicine, Universidade de Marília (UNIMAR), Marília, SP 17525-902 Brazil; 6 Department of Clinical Metabolism, Texas Institute for Kidney and Endocrine Disorders (TIKED), Lufkin, TX 75904 USA; 7 Department of Internal Medicine, University of Texas Medical Branch, Galveston, TX 77555 USA; 8 Department of Research, Research Coordination Center, UNIMAR Charitable Hospital, Universidade de Marília (UNIMAR), Marília, SP 17525-902 Brazil

**Keywords:** Probiotics, Gut-brain-kidney-heart axis, Inflammation, Oxidative stress

## Abstract

The symbiosis between intestinal bacteria and the human body’s physiological processes can modulate health. The intestinal microbiota is linked to the development of neurotrophic factors; therefore, it is increasingly related to the modulation of nervous system pathologies. Moreover, microbiota can interfere with inflammation and oxidative stress, which are closely linked to cardiovascular risk factors and several other inflammatory conditions, such as kidney and neurodegenerative diseases. Probiotics are live microorganisms that help regulate and maintain healthy microbiota; thus, they can help prevent these diseases. Due to these reasons, this review aimed to evaluate the effects of probiotics on the gut, kidneys, brain, and heart homeostasis. Clinical trials showed several positive results with the treatment. In the brain, probiotics reduce depressive symptoms (decreases in HAMA, GAD-7, and BDI-II scales), improving patients’ sleep quality and fatigue, enhancing cognitive subscales while slowing brain atrophy, and reducing IL-6 levels in the central areas, also modulating REM delta power to reduce high-frequency brain waves. Probiotics can also reduce cardiovascular risk factors, such as inflammation. Probiotics can also benefit the heart by decreasing TMAO, LDL-c, TG, CRP, MDA, TNF-α, IL-6, and urea levels, improving dyslipidemia and toxin profiles. Probiotics also increase HDL-c, ApoE, and insulin sensitivity, decreasing BMI, body fat, and the risk of developing chronic hyperglycemia while increasing lean mass. Besides, probiotic supplementation helped reduce toxic uremic toxins (serum urea) and sodium levels, bringing benefits to the kidneys, and improve energy/amino acid metabolism. Probiotics can also modulate and enhance kidney function due to decreased pro-inflammatory TGFβ-1 and TNF-α levels and RUNX2. Furthermore, enhanced gastrointestinal motility and diversity have been reported using specific bacteria. Although probiotics can bring several health benefits, there are still challenges regarding these supplements, such as dose, frequency, and pharmaceutical formula. Therefore, new studies are welcome to deepen the understanding of these microorganisms.

## Introduction

The symbiosis between intestinal bacteria and the human body’s physiological processes can modulate individuals’ health [1–3]. Furthermore, studies have demonstrated that a population’s diet directly affects the colonization of bacteria in the gut [4]. The microbiota is related to diseases outside the gastrointestinal tract, such as the development of nervous system pathologies [5, 6] and cardiovascular and kidney diseases. Therefore, the balanced connection between the intestine, brain, heart, and kidney is fundamental for promoting health [7–9].

Under these conditions, the intestine-kidney, intestine-heart, and intestine-brain axes play a fundamental role in understanding pathologies such as chronic kidney disease (CKD) and cardiovascular diseases [10, 11] and the relationship between these factors [12]. This is due to increased intestinal permeability and the imbalance between intestinal bacteria, which induces the translocation of bacteria and their products into the bloodstream [13–16]. The brain-gut-microbiota axis is also a complex bidirectional [17]. In the connection between the host and microbiota, there is a tangle of neurons named the enteric nervous system (ENS) [18] that modulates the intestine’s motility and secretions, helping defense [19, 20] and helping maintain homeostasis of the gastrointestinal system. For instance, acute interventions, such as the early use of antibiotics by modifying the intestinal microbiota, generate a greater risk of developing psychiatric disorders [21, 22].

In this scenario, probiotics are live microorganisms that help regulate and maintain the host’s health when used in necessary quantities [23–25]. Due to the above, this review aimed to evaluate the effects of probiotics, identifying the primary relationship and interactions between the gut, brain, kidneys, and heart.

## Materials and Methods

### Focused Question

This systematic review was performed to answer the question*: Can probiotics produce beneficial effects on the brain-kidney-intestine-heart axis?*

### Language

Only studies in English were selected.

### Databases

This review has included studies in MEDLINE–PubMed (National Library of Medicine, National Institutes of Health), COCHRANE, EMBASE, and Google Scholar databases. The mesh terms used were intestine-brain-kidney-heart axis, antioxidant or anti-inflammatory, cardiovascular disease, kidney disease, or neurological diseases. The use of these descriptors helped the identification of studies on the interactions between the effects of probiotics on the intestine-brain-kidney-heart axis. We have followed PRISMA (Preferred Reporting Items for a Systematic Review and Meta-Analysis) guidelines [26] to perform the search.

### Study Selection

Abstracts, conferences, letters to editors, grey literature, editorials, case reports, and poster presentations were not included. Moreover, studies not in English were also excluded. The inclusion criteria for this review were only human interventional studies (clinical trials).

### Data Extraction

We did not select a period to perform the search. The retrieved articles are shown in Table 1. The Population, Intervention, Comparison, and Outcomes (PICO) format was used to perform the search.

**Table 1:** Clinical trials show probiotics’ effects in the heart, brain, kidney, and gut axis

Reference	Model/country	Population	Intervention/comparison	Outcomes	Side effects
Probiotics and the brain					
[27]	Single-center, double-blind, placebo-controlled pilot randomized clinical trial/UK	49 patients with a primary diagnosis of MDD and with a HAMD-17. Placebo: 21 female and four male (27.0 years); Probiotic group: 18 female and six male (32.5 years)	Participants were randomized 1:1 to 4 capsules daily of probiotic (Bio-Kult Advanced; ADM Protexin) or matching placebo during 8 weeks	The probiotic group exhibited a greater improvement in depressive symptoms. Participants in the probiotic group experienced a reduction of 1 severity grade on depression rating scales, on average (HAMA and GAD-7)	Nausea and indigestion were observed only in the probiotic group
[28]	Double-blind placebo-controlled randomized study/Japan	One hundred thirty patients were randomized. FAS-Placebo: 35 females and 25 males (78.9 years). Probiotics: 29 females and 26 males (77.2 years). VSRAD-Placebo: 27 females and 20 males (78.5 years). Probiotics: 20 females and 22 males (77.3 years)	The patients received one sachet containing lyophilized powder of *B. breve* MCC1274 (each sachet contained 2 × 10^10^ CFU or more) or a placebo sachet for 24 weeks	The supplementation improved some subscales of cognitive function and suppressed the progression of brain atrophy	No adverse events related
[29]	Randomized double-blind controlled trial/Switzerland	60 patients (47 finalized the trial) with current depressive episodes. 21 used probiotics (39.43 years, 14 were female and 7 were male) and 26 used placebo (38.77 years, 13 were female and 13 were male)	The patients received a probiotic supplement (Vivomixx®) containing eight different strains. The daily dose contained 900 billion CFU/day for 4 weeks	After the intervention, the patients who received the probiotic supplement increased the abundance of the *Lactobacillus*. This increase was not observed in the placebo group. The increased abundance of *Lactobacillus* was associated with diminished depressive symptoms	The side effects were not reported
[30]	Randomized, Double-Blind, Placebo-Controlled Pilot Trial/Taiwan	40 patients self-reported as insomniacs were randomized into: Control (*n* = 19), eight male and 11 female (25.47 ± 4.64 years); PS128 (*n* = 21), five male and 16 female (26.43 ± 5.95 years)	Participants consumed two capsules of either PS128 or a placebo after dinner/30 days	The test group showed a significant reduction in BDI-II scores. In REM, the delta power% of the probiotic group showed a significant borderline increase (day 15). Participants in the probiotic arm presented fewer depressive symptoms and fatigue, less frequent awakening and arousal, and decreased high-frequency brain wave activity	No serious adverse events were reported
[31]	Randomized, double-blind, placebo-controlled parallel study/Korea	122 healthy adults with psychological stress/symptoms of depression/anxiety were randomized. Experimental group: 45 female and 18 male (38.86 ± 10.89 years); Control Group: 38 female and 21 male (37.63 ± 11.04 years)	Patients received two 500 mg capsules of NVP-1704 once a day for 8 weeks	The treatment has beneficial effects on depression and sleep; there was a reduction in serum levels of IL-6. Individuals treated with the probiotics had gut microbiota composition with reduced ratios of *Enterobacteriaceae* to *Bifidobacteriaceae* and *Enterobacteriaceae* to *Lactobacillaceae,* which could be associated with mental health improvements	No serious adverse reactions
Probiotics and the heart					
[32]	Double-blind randomized controlled trial/Bulgaria	Twenty-nine patients with ASCVD history were randomized. Placebo: 15 individuals (58.86 ± 9.38 years); Probiotics: 14 individuals (58.14 ± 7.09 years). 19 patients with a history of myocardial infarction, and eight patients with type 2 diabetes mellitus	Patients were instructed to take a 500 mg capsule of the probiotic *L. plantarum* (consisting of 1 × 10^9^ CFU) twice daily: 1 h after breakfast and 1 h after evening for 12 weeks	A significant decrease in TMAO levels was observed in the group of individuals who received the probiotic	No adverse effects were reported
[33]	Randomized, triple-blind, placebo-controlled, parallel-group clinical trial/Iran	90 patients with heart failure problems (30–70 years) were randomized: Placebo: 45 (39 finalized the trial) individuals; Probiotics: 45 individuals (41 finalized the trial)	Patients received one symbiotic capsule with 500 mg containing strains of *Lactobacillus, Bifidobacterium,* and *Streptococcus thermophilus* daily/10 weeks	There were positive changes in HDL-c levels. TG had a decrease in the intervention group. There were also no substantial changes in sCD163 and sCD/sTWEAK levels	No side effects were reported
[34]	Randomized, triple-blind, placebo-controlled clinical trial/Iran	Seventy-two women with polycystic ovaries were randomized. Placebo: 36 females (26 finalized the trial); 28.38 ± 6.39 years. Probiotics: 36 females (34 finalized the trial); 28.14 ± 5.74 years	Patients receive 2 g of a symbiotic sachet for 12 weeks. The sachets had *Bacillus coagulans*, *Lactobacillus rhamnosus,* and *Lactobacillus helveticus*	Significant decrease in CRP levels; no significant changes in lipid profile compared to placebo	No adverse effects were reported
[35]	Randomized, double-blind, and placebo-controlled trial/China	130 patients with MS aged 30–65 years were randomized: Placebo: 64 individuals; Probiotics: 66 individuals	Patients were instructed to take one capsule per day, at the same time, containing the probiotic *L. paracasei* for 12 weeks	There was an improvement in MS. The intervention improved lipid profile, especially triglyceride-rich lipoproteins. There was an improvement in FMS	No adverse effects were reported
[36]	A randomized single-center, prospective, double-blind, placebo-controlled, parallel-group trial/Thailand	42 patients with hypercholesterolemia were randomized: Placebo: 46.0 ± 5.1 years old and 82.6% female; Probiotics: 48.5 ± 5.3 years old, 65.2% female	Patients used a capsule of strain *L. paracasei* TISTR 2593/daily dose of 350 mg per capsule for 12 weeks	The results showed a reduction in LDL-c, MDA, and TNF-α and increased levels of serum apolipoprotein E	No adverse effects or clinical symptoms
[37]	Randomized, double-blind, placebo-controlled trial/China	60 patients with CAD were randomized: Placebo: 24 individuals; Probiotics: 36 individuals	Individuals in the probiotic group received atorvastatin, metoprolol, and a sachet of Probio-M8 powder per day/6 months	Less TMAO and proatherogenic amino acids were detected in the group treated with the probiotic intervention. Coadministration of Probio-M8, atorvastatin, and metoprolol reduced IL-6 and LDL-c levels; effects were more potent in the Probio-M8 group compared to the placebo group	No adverse effects were reported
[38]	Randomized double-blind controlled trial/Belgium	32 patients with MS were randomized: Placebo: 5 males and six females; 49.45 ± 9.67 years. Probiotics (pasteurized): 4 males and eight females; 52.75 ± 7.16 years. Probiotics (living): 6 males and three female; 52.89 ± 8.59 years.	Participants ingested *Akkermansia muciniphila* (Alive) or pasteurized *Akkermansia muciniphila* daily/3 months. Another group ingested the placebo	The pasteurized form of *Akkermansia muciniphila* resulted in a significant increase in insulin sensitivity, reduced fasting insulin levels, and reduced total cholesterol. LPS levels were significantly reduced in the pasteurized group	Nausea, flatulence, bloating, and cramps
[39]	Randomized double-blind, parallel, placebo-controlled trial/USA, Canada, and Iran	20 ESRD patients undergoing HD were randomized: Placebo: 4 female and seven male (57.6 ± 9 years); Probiotics: 3 female and six male (53.8 ± 11.8 years)	20 g/day of HAM-RS2 (first month) and 25 g/day of HAM-RS2 (second month)	Serum urea, IL-6, TNF-α, and MDA were significantly reduced in the HAM-RS2-treated group. Bacterial analysis presented an increase in the abundance of the *Faecalibacterium* genus in the probiotic-treated group and remained unchanged in the placebo group	No side effects reported
[40]	Randomized controlled clinical trial, double-blind/Italy	48 women with NWO (24%; 40.00 ± 12.56 years), NWL (26%; 30.18 ± 2.04 years), and PreOB/OB (50%; 33.57 ± 10.57 years) were randomized 1:1 into two groups (placebo and intervention)	One group received daily n.1 bag of POS or placebo for 3 weeks. After a three-week washout period, the groups were reversed and received the alternate intervention for an additional three-week period	Treated group showed reduction in BMI, body fat, and increase in free fat mass. Improvement in orocecal transit time was observed, and differences in meteorism and defecation frequency	No adverse effects were reported
Probiotics and the kidney					
[41]	A randomized, controlled, and single-blind clinical trial/Brazil	39 patients with CKD were randomized: Placebo: 5 female and 15 male (64.22 ± 9.68 years). Probiotics: 7 females and 12 males (62.85 ± 11.74 years)	Patients received 100 mL of unfermented probiotic milk with *Bifidobacterium longum* strain and 40 g of extruded sorghum flakes; the control group received 100 mL of pasteurized milk and 40 g of extruded corn flakes for 7 weeks	The symbiotic meal improved energy metabolism and amino sugar metabolism. There were a reduction in BMI, a better synthesis of SCFAs, an improvement in gastrointestinal symptoms, and a reduction of uremic toxins in patients with CKD	No side effects were reported
[42]	A randomized clinical trial, double-blind/Spain	31 patients in HD were randomized: Placebo: 8 male and two female (65.1 ± 18.4 years); Control group: 8 male and three female (76.3 ± 8.7 years); Probiotics: 7 male and three female (66 ± 18.5 years)	The control group received only dietary recommendations. The placebo group received an oral supplement (Renacare®). The probiotics group received the same supplement as the placebo group, plus probiotics/6 months	Increased expression of the microRNAs miR-29a and miR-29b in the probiotic group. Reduction in the expression of TGFβ−1 in the groups that received the probiotics. Reduction in RUNX2 and TNF-α in the group that received probiotics. There was an increase in PTEN in the probiotic group	No adverse effects were reported
[43]	A single-center double-blind randomized clinical trial/Mexico	92 patients, almost half with diabetes, and a quarter had CKD. Most patients had severe AKI. Control group: 22 female and 22 male (58.4 ± 15.9 years); Probiotics: 25 female and 23 male (55.5 ± 16.7 years)	Patients in the intervention group received two capsules of Simbin-RNL® (540 mg capsule containing strains of *Streptococcus thermophilus, Lactobacillus acidophilus*, *Bifidobacterium longum*) or two placebo capsules every 24 h for seven consecutive days	The urea levels decreased in the probiotic group, which was not observed in the placebo group. Furthermore, sodium levels decreased in the probiotic group and increased in the placebo group	Diarrhea, abdominal distension, nausea, vomiting, and rash
[44]	Randomized, double-blind, placebo-controlled study/Brazil and France	33 patients receiving HD were randomized: Placebo: 7 females and 10 males (50.3 ± 8.5 years); Probiotics: 5 females and 11 males (53.6 ± 11.0 years)	Patients received three capsules of probiotics (*Streptococcus thermophilus*, *Lactobacillus acidophilus*, and *Bifidobacterium longum*) or placebo daily/3 months	In the probiotic group, pre-dialysis serum urea and potassium plasma levels increased, and there was a reduction in fecal pH; IS plasma levels increased	No adverse effects were reported
Probiotics and the gut					
[45]	Double-blind, randomized, placebo-controlled trial/China	Two hundred fifty patients (44.5 ± 16.7 years) with functional constipation were randomized. Placebo: 38 females and 12 males (42.2 ± 17.5 years); Group A: 40 females and 10 males (46.9 ± 17.4 years); Group B: 41 females and nine males (45.1 ± 16.8 years); Group C: 40 females and 10 males (45.3 ± 16.5 years); Group D: 34 females and 16 males (43.1 ± 15.6 years)	Group A: polydextrose, oligosaccharides, and resistant dextrin for each sachet; Group B: Psyllium husk, oligosaccharides, and D-mannitol per sachet; Group C: wheat bran, Psyllium husk, oligosaccharides, and resistant dextrin for each sachet; Group D: *Bifidobacterium animalis* subsp. *Lactis*, *Lacticaseibacillus rhamnosus*, oligosaccharides for each sachet; Group Placebo: maltodextrin. Participants were required to take two sachets of warm water (≤ 37 °C) every day	Group D presented a marginal decrease in plasma 5-hydroxytryptamine. Group A presented a higher *Bifidobacterium* count than placebo at weeks 2 and 4. *Anaerostipes* presented increasing trends in groups B and C, which were associated with the BMF increase. *Bacteroides* were positively correlated with BMF	No severe adverse effects were reported
[46]	Randomized, double-blind, placebo-controlled, multicenter clinical trial/Korea	53 healthy elderly individuals were randomized. Placebo: 26 individuals (72 years of average age). Probiotics: 27 individuals (71.11 years of average age)	Participants received placebo or probiotic capsules (strains of *Bifidobacterium bifidum *BGN4 and *Bifidobacterium longum* BORI) for 12 weeks	There was an increase in IPA in the probiotics group, which had a significant relation to gut bacterial profiles. Elevated IPA levels were also associated with the BDNF in the probiotics group. In vitro treatment with IPA reduced TNF-α in activated microglia and neuronal cells	No severe adverse effects were reported
[47]	Randomized double-blind controlled trial/Austria and Ireland	61 patients with depression were randomized. Placebo: 27 females and six males (40.11 ± 11.45 years); Probiotics: 20 females and eight males (43.00 ± 14.31 years)	The probiotic group received a multistrain probiotic plus biotin or placebo/4 weeks. OMNi-BiOTiC® contained *B. bifidum W23, B. lactis W51, B. lactis W52, L. acidophilus W22, L. casei W56, L. paracasei W20, L. plantarum W62, L. salivarius W24* and *L. lactis W19.* In addition, 125 mg D-Biotin, 30 mg of common horsetail, 30 mg of fish collagen, and 30 mg of keratin plus matrix. The placebo included D-Biotin, common horsetail, fish collagen, and keratin + matrix	Both groups improved their psychiatric symptoms. *Ruminococcus gauvreauii* and *Coprococcus 3* were found to be more abundant, and diversity was higher in the probiotics-treated group after 28 days. The results showed a significant difference between the probiotic and placebo groups. The probiotic group differed significantly in beta diversity compared to the placebo. Regarding overall differential abundance, there was an increase in *Ruminococcus* (R.) *gauvreauii* in the interventionprobioti group. Furthermore, an increase in taxonomically related *Coprococcus 3* in the intervention group was observed	No severe effects were reported

*AKI*, acute kidney injury; *ASCVD*, atherosclerotic cardiovascular disease*; BDI-II Scores*, Beck Depression Inventory-II Scores; *BDNF*, brain-derived neurotrophic factor; *BMF*, bowel movement frequency; *BMI*, body mass index; *CAD*, coronary artery disease; *CFU*, colony-forming unit; *CKD*, chronic kidney disease; *CRP*, C reactive protein; , diabetic ketoacidosis; *ESRD*, end stage renal disease; *FAS*, full analysis set; *FMS*, flow-mediated slowing; *GAD-7*, generalized anxiety disorder 7; *HAMD-17*, Hamilton Depression Rating Scale, 17 items; *HAM-RS2*, high-amylose maize resistant starch type 2; *HAMA*, Hamilton Anxiety Rating Scale; *HD*, hemodialysis; *HDL-c*, high-density lipoprotein cholesterol; *IL-6*, Interleukin-6; *IPA*, indole-3-propionic acid; *IS*, indoxyl sulfate; *LDL-c*, low-density lipoprotein cholesterol; *LPS*, lipopolysaccharide; *MDA*, malonaldehyde; *MDD*, major depressive disorder; *MS*, metabolic syndrome; *NVP-1704*, probiotic NVP-1704; *NWO*, normal weight obese; *NWL*, normal weight lean; *POS*, probiotic oral suspension; *PreOB/OB*, pre-obese/obese; *PS128*, probiotic PS128; *PTEN*, phosphatase and tensin homologue; *REM*, rapid eye movement; *RUNX2*, runt-related transcription factor 2; *sCD163*, soluble CD163; *sTWEAK*, soluble TNF-like weak inducer of apoptosis; *SCFAs*, short-chain fatty acids; *TG*, triglycerides; *TGFβ−1*, transforming growth factor beta 1; *TMAO*, trimethylamine N-oxide; *TNF-α*, tumor necrosis factor alpha; *VSRAD*, voxel-based specific regional analysis system for Alzheimer’s disease

## Results

**Fig. 1:**
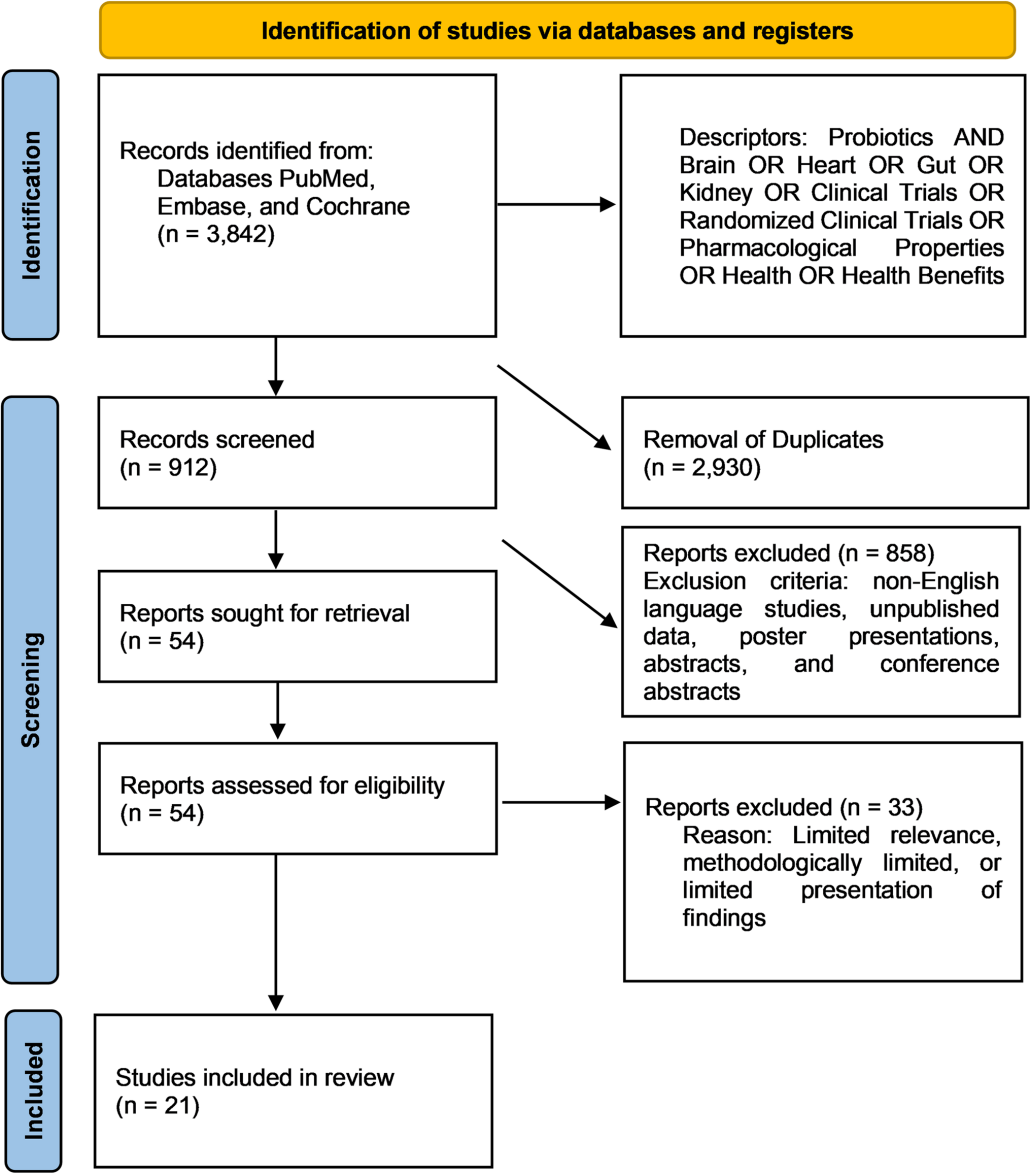
PRISMA flowchart showing the study selection

The study selection is shown in Fig. 1, and the retrieved studies’ results are described in Table 1. Twenty-one articles were included. Nikolova et al. [27] found that the use of the probiotic Bio-Kult Advanced is effective against depression. Protexin® promoted a reduction in depressive symptoms. Schaub et al. [29] visualized increased *Lactobacillus* bacteria in the intestinal microbiota using the probiotic Vivomixx®. Asaoka et al. [28] found benefits and improved cognitive function, preventing brain atrophy with probiotics supplementation. Lai et al. [45] also found positive results in patients with functional constipation. Ho et al. [30] observed decreased high-frequency brainwave activity. Lee et al. [31] demonstrated that probiotics promote positive results regarding the reduction of depressive symptoms and improvement of sleep. Spasova et al. [32] demonstrated positive results with the trimethylamine N-oxide (TMAO) drop. Matin et al. [33] demonstrated improved high-density lipoprotein cholesterol (HDL-c) levels and reduced triglyceride levels. Other authors [34, 35] found positive results in polycystic ovaries and metabolic syndrome. Yang et al. [35] showed that probiotics can improve lipid profiles and metabolic syndrome. Khongrum et al. [36] showed reduced levels of lipoproteins with a high potential for developing fatty plaques. Sun et al. [37] showed that probiotics can lead to a drop in inflammatory substances, such as interleukin-6 (IL-6). Depommier et al. [38] showed increased insulin sensitivity and reduced fasting insulin levels. Laffin et al. [39] found an increase in the proportion of *Faecalibacterium* bacteria. De Lorenzo et al. [40] significantly reduced body mass index (BMI) and body fat and improved orocecal transit time. Lúcio et al. [41] showed reduced uremic toxins in CKD individuals. Sasso et al. [42] observed a reduction of tumor necrosis factor-alpha (TNF-α) in the group that received the supplement. Chávez-Íñiguez et al. [43] showed reduced urea and sodium levels in the intervention group. Borges et al. [44] obtained a reduction in fecal pH. Lai et al. [45] experiment showed a decrease in plasma 5-hydroxytryptamine. Kim et al. [46] found a reduction in the concentration of the pro-inflammatory TNF-α. Reininghaus et al. [47] found that the supplementation promoted a greater abundance of *Ruminococcus gauvreauii* and *Coprococcus 3.*

## Discussion

### Intestinal Microbiome and Its Health Implications

**Fig. 2:**
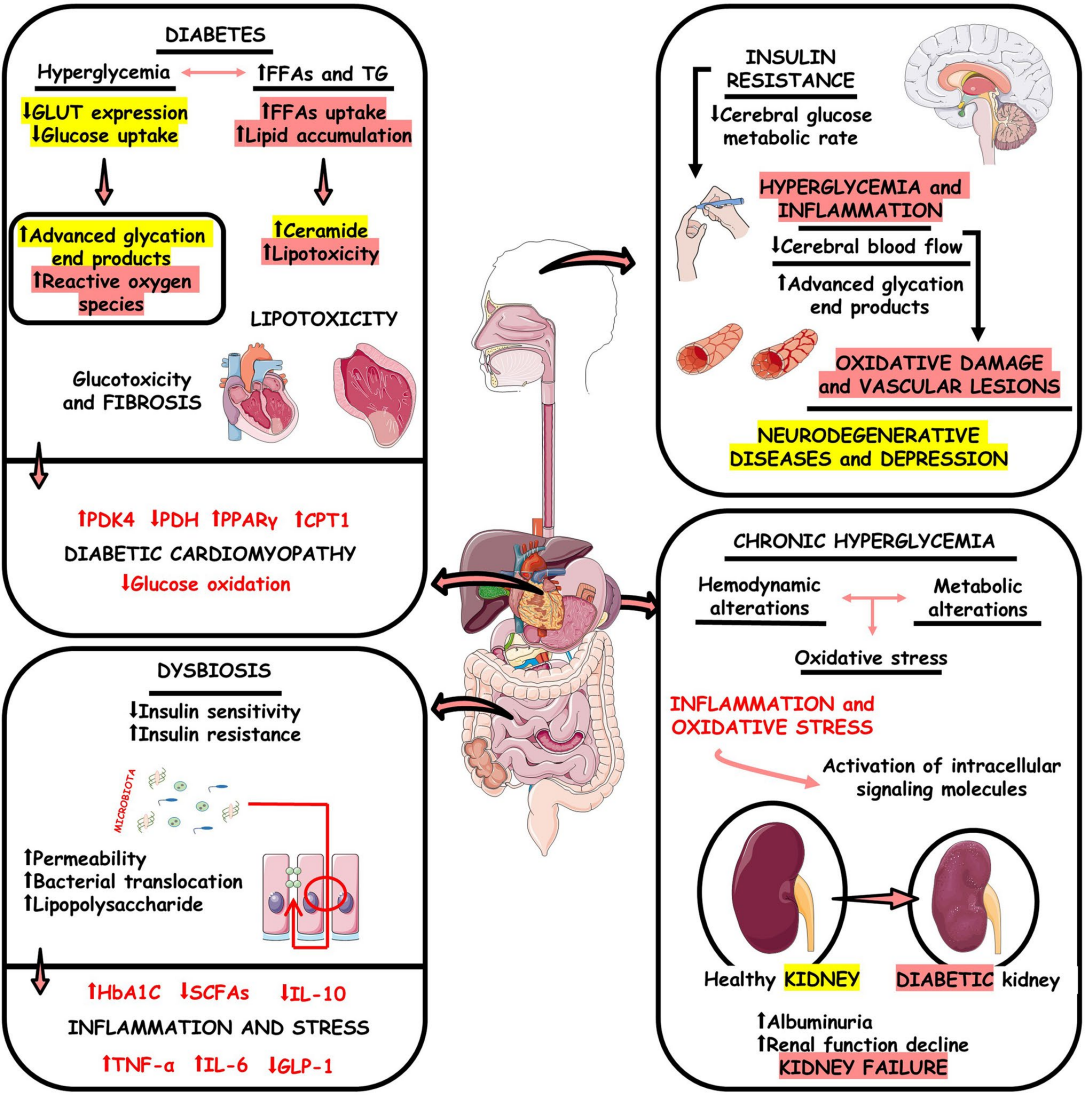
The imbalance of the intestinal microbiota causes a reduction in short-chain fatty acids (SCFAs) and, consequently, in brain neuroplasticity. Furthermore, chronic and acute stress causes intestinal inflammatory disorders. In addition, traumatic brain injury (TBI) interferes with renal homeostasis. It causes acute kidney injury (AKI), and, in this relationship between the kidney and brain, the dysregulation of metabolism in chronic kidney disease (CKD) promotes dementia. Furthermore, intestinal bacterial imbalance promotes CKD progression and impaired intestinal barrier function. Furthermore, the imbalance in brain natriuretic peptide (BNP) produced by the heart interferes with kidney diseases, and the stenosis that CKD or AKI can cause promotes an increase in blood pressure by the renin–angiotensin–aldosterone system (RAAS). Additionally, the unbalanced intestine promotes the release of trimethylamine N-oxide (TMAO), which contributes to the proliferation of atheroma plaques that lead to heart failure, and, in a double pathway, heart failure is associated with a lack of intestinal motility. Downwards arrow (↓), decrease; Upwards arrow (↑), increase; FFAs, free fatty acids; TG, triglycerides; GLUT, glucose transporter; PDK4, pyruvate dehydrogenase kinase 4; PDH, Pyruvate Dehydrogenase; PPARγ, peroxisome proliferator-activated receptor gamma; CPT1, carnitine palmitoyltransferase 1; TNF-α, tumor necrosis factor-alpha; IL, interleukin; GLP-1, glucagon-like peptide one; HbA1c, glycated hemoglobin

The intestinal microbiota is vital for maintaining the body’s homeostasis [48] and has several implications for the consolidation of health. *Lactobacillus* spp., *Bifidobacterium* spp., *Streptococcus* spp., *Enterococcus* spp., and *Saccharomyces boulardii* are the most commonly administered strains for supplementation [49]. Studies have shown that using multiple strains of probiotics or a single strain has the same benefit in microbiota [50]. Some concerns have also been raised about the presence of unwanted live microbial contaminants in the supplement [51]. This is because probiotics are created to be administered alive, so they do not undergo sterilization [52]. Figure 2 shows the general effects of metabolic alterations in the brain-gut-kidney-heart axis.

Cardiovascular-kidney-metabolic (CKM) syndrome is characterized by several hemodynamic and neurohormonal processes that interact with each other, including the renin–angiotensin–aldosterone system (RAAS), sympathetic hyperactivity, and chemical mediators (nitric oxide, prostaglandins, endothelins, etc.) and oxidative stress [53–56]. In this sense, the dysregulation of metabolism is directly related to the development of kidney diseases since hyperglycemia causes an exacerbated increase in the entry of glucose into cells, which leads to the production of mitochondrial superoxide, resulting in oxidative stress and inflammation [57, 58]. The increase in reactive oxygen species (ROS) causes tissue damage, and for example, the activation of the polyol and hexosamine pathways generates advanced glycation end products (AGEs). These molecules result from the non-enzymatic glycation of proteins and the upregulation of their cellular receptor [59]. AGEs can directly damage the heart, blood vessels, kidneys, and other organs. They are known to be associated with hypertension and may cause cardiovascular and kidney disease. One mechanism is the accumulation of AGEs, causing glomerular hypertrophy and injury [60], which results in proteinuria and eventual end-stage renal disease [61]. The kidneys may also play a role in AGE metabolism, as they can be taken up and catabolized by the renal proximal tubule [62]. Further, AGEs cause apoptosis of renal proximal cells and inflammatory, thrombotic, and fibrotic changes [63]. Furthermore, hyperglycemia is associated with activating the local RAAS in the myocardium and kidneys, which can promote vasoconstriction (increasing blood pressure), fibrosis, and worsening organ dysfunction [64]. Hyperglycemia increases the brain’s susceptibility to lipopolysaccharide-induced neuroinflammation in the nervous system by reprogramming astrocytes [65, 66]. Furthermore, AGEs accumulate in the brain, leading to neurodegenerative conditions, such as Alzheimer’s disease (AD). This pathology is influenced by receptors for AGEs and the toll-like receptor 4 (TLR4), which results in the glycation of proteins that generate irreversible AGEs. The main reaction is Maillard, which causes abnormal glucose metabolism in the brain, oxidative stress, malfunction of mitochondria, neuronal plaque deposition, and death [67–69]. It is also noted, as well, that in diabetic nephropathy (DN), the supplementation with *L. acidophilus* strains, *B. longum*, and *B. bifidum* may help reduce blood urea level, serum creatinine, and blood glucose. Furthermore, protein in urine was reduced significantly. There was an improvement in blood pressure and glucose tolerance in the mouse model [70, 71].

One mechanism that demonstrates the intimate contact between the heart and the kidneys is the RAAS, which controls extracellular fluid volume and blood pressure [72]. The imbalance of this mechanism gives rise to cardiovascular and kidney diseases [73]. Renin is a proteolytic enzyme synthesized in its inactive form (prorenin) by the juxtaglomerular cells of the kidney. In response to reduced sodium supply, reduced pressure in the afferent arterioles of the glomerulus, and sympathetic activation, prorenin is released into the bloodstream, where it is converted to active renin, which is responsible for the conversion of angiotensinogen to the decapeptide angiotensin (Ang) I [74]. Angiotensinogen is produced mainly by hepatocytes. Angiotensin-converting enzyme (ACE) hydrolyzes Ang I to Ang II. The relationship between the kidney and the RAAS is well known and involves the control of blood pressure, fluid homeostasis, and electrolyte balance [75]. ACE2 is widely expressed in the kidney, and altered ACE2 distribution is associated with renal disease [76]. Furthermore, the RAAS also plays a vital role in maintaining cerebral homeostasis, as high blood pressure can cause damage to the brain in the form of hypertensive encephalopathy, ischemic stroke, and intracerebral hemorrhage. Furthermore, hypertension causes chronic changes in brain tissue that will manifest as impaired brain functions, including cognitive impairment [77]. Cardiovascular diseases and kidney diseases are highly interconnected, and the dysfunction of one organ causes the failure of the other, leading to the failure of both organs, causing cardiorenal syndrome [78–80].

The brain-gut-kidney-heart relationship is evident in various functions and irregularities of the body. Among the main substances present in the heart are the cardiokines. The main hormonal types are the atrial natriuretic peptide (ANP) and the B-type natriuretic peptide (BNP), which have an analogous protein structure formed by a peptide ring with a cysteine bridge [81, 82]. The cardiomyocytes are responsible for secreting in response to cardiac changes, such as strain and ischemia, and also play a crucial role in cardiorenal protection [83], so BNP levels represent the balance of cardiac generation and renal clearance [84, 85]. BNP also has a vital renoprotection effect, as it inhibits sodium reabsorption in the nephron and improves the glomerular filtration rate (GFR) and renal plasma flow (RPF), inhibiting multiple plasma vasoconstrictors [86]. Additionally, higher BNP levels may indicate heart failure or other conditions related to the circulatory system [87, 88]. Another crucial substance in this connection between the heart and the kidney is ANP. It has been proposed to play a role in susceptibility to hypertension. Elevated ANP levels have been considered a hallmark of heart failure [89] and nephrotic syndrome [90]. ANP, encoded by the NPPA gene, is a hormone that promotes salt excretion, vasodilation, and blood pressure reduction [91]. Moreover, ANP expression is induced by elevated atrial pressures [92], while BNP is secreted in response to a reflex of ventricular overload [93]. The kidney represents the primary target organ of these cardiokines, and they stimulate diuresis and natriuresis. Thus, ANP and BNP promote vasodilation [94]. The brain communicates directly with the heart through the parasympathetic pathway (involving vagal motor neurons in the brainstem and postganglionic neurons in the heart) and the sympathetic pathway (involving preganglionic neurons) [95]. Parasympathetic pathways modulate adverse chronotropic effects through cholinergic signaling, whereas sympathetic outputs positively mediate chronotropy and inotropy through adrenergic signaling [96]. Furthermore, the heart plays a role in synthesizing and releasing oxytocin, commonly called the “love” or “social bonding” hormone. Oxytocin is implicated in various cognitive processes, such as tolerance, trust, and social bonding [97]. Another common link, in addition to the hormonal release, is the rostral ventrolateral medulla (RVLM), which is the final common efferent pathway for increased blood flow, which derives from inotropic and chronotropic cardiac responses and peripheral arterial and venous constriction [98]. In contrast, the dorsal vagal nucleus may receive input from the nucleus *tractus solitarius* (NTS) and promote parasympathetic signaling to the heart [99]. In summary, the relationship between the brain and the heart is fundamental for the body’s homeostasis and physiological response [100].

A close relationship is also observed between the intestine-kidney-heart axis [101]. This is because the development of some strains in the intestinal microbiota is responsible for certain cardiovascular complications [102]. Alterations in the gut microbiome have favored the conversion of dietary choline to TMAO, a toxic circulating biomarker that correlates with atherosclerotic lesions [103]. Furthermore, intestinal dysbiosis, which is the alteration of the intestinal microbiota, could aggravate atherosclerotic progression by reducing the abundance of short-chain fatty acids (SCFAs), acetate, propionate, and butyrate, which generally act as inhibitors of vascular inflammation [104]. Another link between the heart and the intestine, in pathological form, is inflammation and myocardial fibrosis, which are the basic pathological mechanisms of heart failure. With heart failure, there is a lack of intestinal motility, impaired absorption, and damage to the mucosal barrier of the intestinal epithelium, which increases intestinal permeability and leads to failure in the prompt removal of bacteria and food debris [105, 106]. This promotes the proliferation of Gram-negative bacteria, such as *Klebsiella*, resulting in the easy entry of toxic mediators. Furthermore, the more severe the heart failure, the higher the phenylacetylglutamine level, which can be used as an indicator for early screening, diagnosis, and treatment for congestive heart failure [107].

There is a direct association between the kidneys and the gut since changes in the intestinal microbiome contribute to the progression of CKD [57]. One of the complications of CKD is impaired intestinal barrier function [108]. Fortunately, recent studies have shown that improving microbiota composition can reduce uremic toxins and immune inflammation in patients with kidney disease [109]. The use of oral agents that absorb intestinal toxins and symbiotics that help improve the composition of the microbiota results in improved kidney function in people with CKD [110, 111].

The kidneys play a fundamental role in the proper functioning of the brain. In this aspect, some kidney diseases can directly affect the functioning of the brain; CKD is a strong risk factor for developing dementia [112, 113]. Dementia is a syndrome characterized by deterioration in multiple cognitive domains [114]. A potential treatment or prevention of nervous disorders has been analyzed, as kidney dysfunction has been closely associated with several diseases that affect the central nervous system (CNS) [115]. Previous studies have suggested proteinuria levels and acute kidney injury (AKI) associations with the risk of cognitive impairments [116]. Furthermore, patients with traumatic brain injury (TBI) are at risk of developing AKI, which can worsen the patient’s prognosis [117]. There is also a close relationship between BNP releases in patients with TBI [118].

In addition to all these connections, communication between the brain and the gut has shown increasing promise for treating diseases [119]. Moreover, neuronal pathways physically link the gut and the brain. The chief is the vagus nerve [120], which extends from the brainstem to innervate the gut and the ENS [121]. As an example of direct signaling, SCFAs are lipids produced by gut microorganisms through fermentation of dietary fiber that can act on the CNS [122] by controlling neuroplasticity, epigenetics, and gene expression and the immune system in preclinical models [123]. The microbiota can affect host appetite and feeding behaviors by modulating the production of endocrine signals from enteroendocrine cells (EECs) in the intestinal epithelium [124], including the production of the hormone glucagon-like peptide 1 (GLP1) [125] which occurs because hunger can be modulated by GLP1 secreted by colonic enteroendocrine L cells, in response to the bacterial metabolite indole that stimulates colonic vagal afferent activity [126]. Furthermore, microorganisms that inhabit the intestine can synthesize neurotransmitters themselves and induce neurotransmitter production by their hosts [127]. Changes that alter the gut microbiota can activate pro-inflammatory cytokines [128] and increase intestinal permeability, leading to the development of insulin resistance associated with AD [129, 130].

**Fig. 3:**
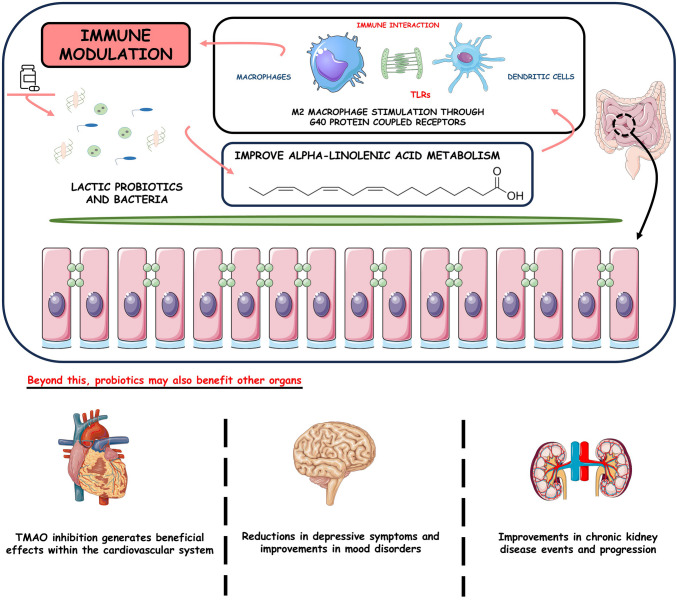
Probiotics can modulate immune response and help maintain the integrity of intestinal cells. Moreover, they can protect the cardiovascular system and brain and improve kidney disease progression. TLRs, toll-like receptors; TMAO, trimethylamine N-oxide

Given all these factors, the intrinsic relationship and dependence of the gut-kidney-heart-brain axis are observed. In this aspect, dysbiosis, that is, the alteration of the intestinal microbiota, modifies the entire homeostasis [131], contributing to the development of depression [132] by behavior through abnormal synapse pruning in microglia mediated by complement C3 [133] that causes a worsening of heart and kidney diseases, such as CKD. In this sense, the use of probiotics, in addition to contributing to improved gastrointestinal function [134], can also be used to prevent brain and kidney disorders, coronary heart disease, and stroke (Fig. 3) [135].

Despite the multiple pathways presented in this article, including SCFAs, TMAO, and vagal nerve signaling, which are implicated in host-microbiome interactions, the current amount of evidence is insufficient to compare their relative influence directly. Given this, future studies must incorporate mechanistic or interventional designs aimed at disentangling the specific roles and hierarchical importance of the pathways above to depict more nuancedly their influence on the gut-brain–heart-kidney axis.

### Probiotics

Probiotics are defined as microorganisms that, when administered in the correct form and quantity, bring benefits to the patient [136–138]. They are mainly Gram-positive bacteria, such as *Lactobacillus *and *Bifidobacterium*, capable of competing with harmful microorganisms that could colonize the intestine [139], so they are increasingly emphasized as a contributor to the improvement of various diseases [140]. As such, some probiotics can mitigate the effects of increased intestinal permeability, inflammation, and nutrient malabsorption [141]. Furthermore, SCFAs and other byproducts produced by probiotics can interfere with and inhibit the inflammatory response, thus reducing damage to neurons [142]. Besides, probiotics can modulate gut microbiota and gut-derived metabolites to alleviate CKD progression [143], and some probiotics have been shown to possess a reduced capacity to synthesize gut-derived trimethylamine and therefore TMAO, thereby suggesting that inhibition of TMAO is a factor mediating the cardiac beneficial effects of probiotics [144, 145].

Furthermore, the adhesion of probiotics to epithelial cells can trigger a signaling cascade, leading to immunological modulation [146]; this is evidenced in the action of intestinal lactic acid bacteria that improve the metabolism of α-linolenic acid and induce the differentiation of M2 macrophages through a receptor coupled to G protein 40 [147]. Probiotic bacteria can also aggregate to form a protective barrier that prevents the proliferation of pathogens across the cellular epithelium [148]. In recent research, the effects of probiotics are attributed to a reduction in depression symptoms through supplementation. Segmented filamentous bacteria may explain this increased susceptibility to depression by promoting the production of T helper 17 (Th17) cells. In this case, probiotics may be helpful due to their role as natural competitors [149].

### Clinical Trials

Many studies have shown the benefits of probiotics for the heart, kidneys, brain, and gut axis. Table 1 summarizes the findings of these trials. Table 2 depicts the bias assessment of the randomized studies.

Nikolova et al. [27] conducted a study to evaluate the use of probiotics in reducing depressive symptoms. Using the probiotics Bio-Kult Advanced and ADM Protexin promoted a reduction in depressive symptoms. However, adverse effects such as nausea and indigestion occurred only in the probiotic groups. Besides, this study presents several limitations. Firstly, the authors could not ascertain whether the impact of the utilized intervention was specific to its interaction with the selective serotonin-reuptake inhibitors (SSRIs) or not, being at this point generalizable to other treatments. On the other hand, the authors evaluate the capsule count to assess the patients’ adherence to the proposed treatment, which can lead to overreporting. Finally, the authors did not appropriately randomize the included participants, compromising the findings’ generalizability.

**Table 2:** Bias assessment of the randomized studies following the COCHRANE handbook for systematic reviews and interventions [150]

Study	Question focus	Allocation blinding	Appropriate randomization	Double-blind	Losses (< 20%)	Prognostic and demographic characteristics	Outcomes	Intention to treat analysis	Sample calculation	Adequate follow-up
[27]	Yes	Yes	No	Yes	Yes	Yes	Yes	Yes	NR	Yes
[29]	Yes	Yes	Yes	Yes	No	Yes	Yes	Yes	Yes	Yes
[28]	Yes	Yes	NR	Yes	Yes	Yes	Yes	Yes	Yes	Yes
[30]	Yes	Yes	NR	Yes	Yes	Yes	Yes	Yes	NR	Yes
[31]	Yes	Yes	Yes	Yes	No	Yes	Yes	No	Yes	Yes
[32]	Yes	Yes	No	Yes	Yes	Yes	Yes	No	NR	Yes
[33]	Yes	Yes	Yes	Yes	Yes	No	Yes	No	Yes	Yes
[34]	Yes	Yes	Yes	Yes	Yes	Yes	Yes	No	Yes	Yes
[35]	Yes	Yes	Yes	Yes	Yes	Yes	Yes	Yes	Yes	Yes
[36]	Yes	Yes	Yes	Yes	Yes	Yes	Yes	No	Yes	Yes
[37]	Yes	Yes	NR	Yes	No	Yes	Yes	NR	NR	Yes
[38]	Yes	Yes	Yes	Yes	No	Yes	Yes	No	NR	Yes
[39]	Yes	Yes	NR	Yes	NR	No	Yes	NR	NR	Yes
[40]	Yes	Yes	NR	Yes	Yes	Yes	Yes	Yes	NR	Yes
[41]	Yes	Yes	NR	No	No	Yes	Yes	No	Yes	Yes
[42]	Yes	Yes	Yes	Yes	No	Yes	Yes	No	NR	Yes
[43]	Yes	Yes	Yes	Yes	Yes	Yes	Yes	Yes	No	Yes
[44]	Yes	Yes	No	Yes	No	Yes	Yes	No	NR	Yes
[45]	Yes	Yes	NR	Yes	Yes	Yes	Yes	Yes	Yes	Yes
[46]	Yes	Yes	Yes	Yes	No	No	Yes	No	NR	Yes
[47]	Yes	Yes	Yes	Yes	No	Yes	Yes	No	Yes	Yes

*NR*, not reported

Schaub et al. [29] analyzed the use of the probiotic Vivomixx® in individuals diagnosed with depression. The study revealed an increase in *Lactobacillus* in the intestinal microbiota and a decrease in depressive symptoms. The inhomogeneous gut microbiota may also result from the various probiotic strains utilized during the study’s intervention. Furthermore, the study was conducted for only 4 weeks, including a relatively small sample size, and the authors did not assess the interactions between the probiotics and the general antidepressant medications used in synergy by the included patients, limiting the generalizability of the reported results. In addition, the authors had losses above 20%, limiting the generalizability of the findings and decreasing the confidence in the results.

Asaoka et al. [28] reported a study with 130 patients who ingested a probiotic containing *B. breve* MCC1274. The study had a long period of intervention, lasting 24 weeks. In addition to showing benefits in improving cognitive function and preventing brain atrophy, another benefit was the absence of adverse events. The main limitation of this study is that the researchers used a neuropsychiatry scale at 8 and 16 weeks after baseline to assess this domain, which could not necessarily demonstrate acute changes in behavior. Additionally, these authors did not report information regarding the randomization process of this study, which can raise possible bias due to manipulation of intervention and placebo groups.

Ho et al. [30] analyzed PS128 supplement capsules in individuals with insomnia. Positive results, such as decreased high-frequency brain wave activity, were observed. However, the study was only conducted for 30 days and with a few patients. This study has some limitations. Firstly, the researchers have not identified the intestinal microbiome analysis for the included participants, so they do not know if the probiotic significantly colonized the intestine of the participants. In addition, recruiting only young participants may not fully generalize the results to the overall population. This study did not report information regarding the randomization process or sample calculation, which can limit the generalizability of the findings and raise concerns about intervention and placebo group manipulation.

Lee et al. [31] examined the use of probiotics in adults experiencing stress, depression, and anxiety. They found a reduction in depressive symptoms and an improvement in sleep. Furthermore, the study was carried out for 8 weeks with 122 individuals. There were no severe adverse reactions. However, it is worth noting that the study has several limitations. Firstly, they performed per-protocol analysis, which can induce an overestimation of the proposed treatment’s efficacy and an underestimation of the adverse treatment reactions. Secondly, the researchers did not analyze the patients’ gut microbiota before the intervention, which suggests that the post-intervention analysis may not fully represent the effects of the intervention. Additionally, these authors had losses above 20% in their participants and did not use intention-to-treat analysis. Therefore, essential data could have been missed, which could improve or not the results according to the effects in those who discontinued the intervention.

Spasova et al. [32] analyzed the use of probiotics in patients with atherosclerosis. Under this bias, the study obtained good results with the drop in TMAO. Although the study lasted 12 weeks, the number of patients was low, with only 29 individuals. Additionally, this was a single-center study, which limits the generalizability of the findings. Finally, dietary status throughout the intervention was not carried out, which may limit the understanding of the researchers about the oxidative and inflammatory statuses of the treated patients based on TMAO analyses. Additionally, the authors did not report their randomization process, limiting the interpretation of the findings due to possible group bias. Furthermore, they did not use the intention-to-treat analysis, which can miss essential data from those who discontinued the intervention, limiting the reporting of the results. Finally, they did not report the use of sample calculation methods, which can limit the generalizability of the results due to non-confirmatory grouping.

Matin et al. [33] studied 90 patients over 10 weeks with capsules of probiotic bacteria strains. The main results of the research showed an improvement in HDL-c levels and a reduction in triglycerides. In addition, there were no significant adverse effects. However, due to financial constraints, the researchers could not analyze the gut microbiota composition before, during, and after the intervention. In addition, they did not report on the prognostic and demographic characteristics of the sample studied in their main article. Additionally, they did not use intention-to-treat analysis, probably missing essential data from those who discontinued the intervention. This is particularly problematic because the missing results could benefit or degrade the intervention outcomes.

Hariri et al. [34] analyzed the effect of probiotics on women with polycystic ovaries. Despite only including 72 women, the results were positive, with no significant adverse effects. In addition, the study did not assess intention-to-treat analysis, which could limit the generalizability of the findings based on the missing data. Despite this limitation, the triple-blind nature of this study must be considered a strength. Although the authors did not use intention-to-treat analysis to report their findings, their sample size did not diminish substantially during the intervention.

Yang et al. [35] began research into the effect of the probiotic *L. paracasei* on patients with metabolic syndrome. The study included 130 patients and obtained significant results in improving the lipid profile. Furthermore, there were no significant adverse effects. Besides these positive effects, the sample comprised more than 70% of men, compromising the findings’ generalizability to the female population. This study utilized intention-to-treat analysis to report the findings.

Khongrum et al. [36] analyzed the effect of probiotics on patients with hypercholesterolemia. The study obtained good results in reducing the levels of lipoproteins with a high potential for developing fatty plaques. Despite being carried out for 12 weeks and having no significant adverse effects, few patients were included, only 42 patients. However, this study possesses some limitations. Firstly, they did not assess colonization in feces, which can limit the interpretation of the findings since colonization is an essential indicator of treatment success with the probiotic *L. paracasei* TISTR 2593. In addition, the included participants’ diet, lifestyle, mood, and physical activity have not been monitored during the intervention, and significant acute modifications in these parameters may lead to alterations in intestinal health and limitation of probiotic activity. Finally, they did not use intention-to-treat analysis to report their findings, which can be considered biased.

Sun et al. [37] researched probiotics’ effects on coronary artery disease (CAD) patients. The study, which was carried out over six months with just 60 patients, showed a drop in inflammatory substances, such as IL-6. Furthermore, there were no reported adverse effects. However, the study comprised a relatively small sample size and did not report intention-to-treat analyses, which can limit the generalizability of the reported outcomes. In addition, the author did not report their randomization method and did not report calculation of the sample size, which can be considered biased.

Depommier et al. [38] analyzed supplementation with *Akkermansia muciniphila* in overweight and obese human volunteers. The study was conducted with only 32 patients for 3 months. There were several positive effects of *Akkermansia muciniphila* supplementation, such as increased insulin sensitivity and reduced fasting insulin levels. However, the number of patients enrolled in the randomization does not allow a concrete result. Additionally, the researchers did not find sufficient visceral adiposity or BMI changes within the studied sample; they did not utilize specific and accurate methods to precisely estimate visceral versus subcutaneous fat mass, including dual-energy X-ray absorptiometry. Furthermore, the included participants’ physical activity level and calorie intake were not determined precisely. The intervention also led to adverse effects, which were substantially more gastrointestinal, such as nausea, flatulence, bloating, and cramps. Other limitations can be the losses above 20%, the lack of intention-to-treat analysis, and the lack of sample size calculation reporting, which can be considered biased.

Laffin et al. [39] evaluated the results of amylose-resistant starch (HAM-RS2) supplementation in patients with end-stage renal disease. This microbial analysis showed an increase in the proportion of *Faecalibacterium* bacteria, which play an essential role in the intestine as commensal bacteria. That said, patients experienced a reduction in serum urea, IL-6, TNF-α, and malondialdehyde (MDA). Besides, there was a trend toward an increase in *Prevotella*. The study was conducted with only 20 patients for 2 months. Caution is needed because the researchers did not mention sample calculation or intention-to-treat analyses. Additionally, the researchers did not present a complete table with the demographic and prognostic characteristics of the studied individuals and did not report possible losses, which raises concerns about the generalizability of the findings to the overall population.

De Lorenzo et al. [40] evaluated the possibility of psychobiotic intake modulating the psychological profile and body composition of women affected by normal weight and obesity syndrome. The evaluation was carried out with 48 women. The results included a significant reduction in BMI and body fat, greater body hydration in both groups, and improved orocecal transit time. However, caution is needed because the sample size was too small to predict generalizable results for the overall female population. In addition, the age range was 30 to 40, which only determines results for adulthood and not for elderly women. Besides, they did not report sample size calculation, and the authors did not describe the randomization process in detail, which could have been a potential bias in interpreting the results.

Lúcio et al. [41] researched probiotics in milk for 7 weeks. The study included individuals with CKD and obtained good results, especially in reducing uremic toxins. Despite not reporting adverse effects, only 39 patients were involved in the research. The main limitations of this study are the lack of control groups receiving *Bifidobacterium longum* and the use of long-term dialysis patients, which can limit the generalizability of the reported outcomes to the overall population. In addition, the intervention time was relatively short. Finally, the randomization process was not described, the study was not double-blinded, the losses are greater than 20%, and the intention-to-treat analysis was not utilized to report the findings of all participants, including those who discontinued the intervention.

Sasso et al. [42] analyzed probiotics in patients undergoing hemodialysis. The main result reported was the reduction of TNF-α in the group that received the supplement. The study was carried out for 6 months with only 31 patients. However, caution is warranted while assessing the included outcomes due to the small sample size and the massive loss of participants during the study’s intervention, which limited the finding of significant changes in other circulating micro ribonucleic acids (miRNAs). Additionally, the authors did not utilize intention-to-treat analysis or report calculation of the sample size, limiting the generalizability of the findings.

Chávez-Íñiguez et al. [43] observed the use of Simbin-RNL® in patients with diabetes, CKD, and AKI. The intervention group had reduced urea and sodium levels. The study included 92 patients and reported several side effects, such as diarrhea, abdominal bloating, and vomiting. Another adverse effect included rash occurrence. The study period was reduced to 7 days. However, the sample size was relatively small due to the lack of sufficient previous information to calculate the sample size accurately. In addition, there was a lack of measurements of the intestinal microbiota in the participants’ stool, systemic inflammation outcomes and parameters, and biomarkers of renal tubular damage, which could have reflected kidney damage more appropriately.

Borges et al. [44] conducted a 3-month study to evaluate the action of probiotic capsules in hemodialysis patients. The study involved only 33 patients. The results obtained included a reduction in fecal pH. However, the small sample size and the lack of alimentary statuses of the included patients might limit the generalizability of the outcomes. Furthermore, no sample size calculation or intention-to-treat analysis were reported, and the losses were substantial.

Lai et al. [45] evaluated the use of probiotics distributed in groups A, B, C, D, and P. A total of 250 patients were included. The experiment showed a reduction in plasma 5-hydroxytryptamine. Group A resulted in a higher *Bifidobacterium* as the main result. Furthermore, no adverse effects were reported. However, caution is needed to assess the results of this study. Firstly, it is worth noting that the authors did not subcategorize patients for constipation statuses. Therefore, they could not detect whether the intervention could affect different constipation types, such as those with dyssynergic defecation, who might not directly respond to interventions involving dietary supplements with fiber or probiotics. In addition, the authors could not detect metabolic interactions between host and gut microbiomes due to the lack of microbiota metabolite data. Finally, the included participants had a wide range of age representation, which could interfere with the individual variability of the findings on the gut microbiota alterations.

Kim et al. [46] examined probiotics in healthy elderly individuals. The main result was a reduction in the concentration of pro-inflammatory TNF-α. Despite being carried out with only 53 individuals, the study lasted 12 weeks and reported no severe adverse effects. However, this study has some limitations, including that it does not report data on functional insights about the gut microbial profiles due to the lack of ribosomal ribonucleic acid (rRNA) data. Additionally, the intention-to-treat analysis was not utilized in reporting findings, limiting the generalizability of the included outcomes. Finally, the sample size calculation was not reported, and the demographic and prognostic characteristics of the included subjects were not disclosed.

Reininghaus et al. [47] initiated a study to evaluate the use of probiotics in patients with depression. The research was carried out with 61 individuals for just 4 weeks. The most notable results were the greater abundance and diversity of *Ruminococcus gauvreauii* and *Coprococcus* 3. Furthermore, no serious adverse effects were reported. The small sample size and the massive drop-out from the study may be considered limitations of the included outcomes. Additionally, the study did not utilize the intention-to-treat analysis technique, which limits the generalizability of the findings by missing data from those who discontinued the intervention protocol.

### Take-Home Messages for Clinicians and Future Endeavors

The ideas discussed in the above sections, besides being preliminary, give important insights about how clinicians may use probiotics in their daily routines to combat brain, heart, kidney, and even intestinal diseases and disorders. Modulating the gut-brain axis may be central to psychiatry and primary care interventions for mental health support. The gathered data suggests that probiotics may be an efficient adjunct therapy for depression and anxiety in patients with mild to moderate depression or anxiety who are already receiving medication. Probiotics can also be used in these patients to improve symptoms like insomnia or fatigue when unresponsive to sleep hygiene alone, as probiotics have been shown to improve rapid eye movement (REM) quality, delta wave activity, and reduce night arousals. However, since probiotics are not a standard therapy for these issues, it is worth noting that clinicians must persevere in monitoring their patients continuously with standardized scales, including Beck Depression Inventory-II (BDI-II) and Generalized Anxiety Disorder 7 (GAD-7). Future research will be able to validate the current observations and potentially reveal novel probiotic strains or strain combinations that yield more pronounced or better results in psychobiological health.

Against kidney diseases, probiotics were found to reduce uremia, improving ammonia metabolism for patients in the middle-to-final stage of renal disease. Therefore, clinicians could consider using probiotics as part of nutritional counseling in patients undergoing treatment for CKD. Prescribing probiotics in these patients may also improve dialysis patients’ quality of life by ameliorating symptoms like bloating and constipation, as well as the fecal pH, aiming for better gut-derived toxin clearance and comfort. However, since probiotics are not a standard therapy against CKD or its associated symptoms, it is essential to note that clinicians, if prescribing probiotics for CKD patients, must elucidate their treatment lines with serum urea, C reactive protein (CRP), pro-inflammatory cytokines, and electrolytes screening to assess broadly their systemic response and adjust dose or probiotic strain if needed. If larger, better-designed clinical studies with more substantiated findings become available, probiotics could be utilized as standard adjunct therapy against kidney diseases, improving health conditions and reshaping how we approach CKD.

In cardiology, probiotics have an even more pronounced effect on cardiometabolic health. Probiotics improved lipid content and effectively managed inflammatory markers in patients with cardiometabolic conditions. Therefore, probiotics may be helpful to counter low-density lipoprotein cholesterol (LDL-c) and increase HDL-c in some patients as an adjunct to lifestyle changes or standard medications. In this scenario, probiotics were found to be an effective supplement for metabolic syndrome, as they were also found to cause BMI reduction and control fat mass, improving insulin sensitivity and glycemic control. For patients with a higher risk of atherosclerosis, probiotics can also be used to reduce TMAO levels, particularly in those with high meat intake, leading to reduced atherosclerosis, which, in long-term assessments, will reduce cardiovascular events and outcomes. Table 3 describes the probiotic strains of the included studies and their main health effects driving the gut-brain-kidney-heart axis.

**Table 3:** Probiotic strategies based on probiotic strains included in the studies

Probiotic strain	Health effects related to the gut-brain–heart-kidney axis
Bio-Kult Advanced (multi-strain)	Improvement of depression
*Bifidobacterium breve*	Improve cognitive function and suppression of brain atrophy in the central nervous system
Vivomixx® (multi-strain)	Improved depressive symptoms due to increased *Lactobacillus* content
*Lactobacillus plantarum*	Reduced TMAO levels in the cardiovascular system
NVP-1704 (*Lactobacillus reuteri* and *Bifidobacterium adolescentis*)	Improved depressive symptoms and sleep quality, reduction of IL-6, and beneficial effects through gut microbiome shifts in the central nervous system
*Lactobacillus*, *Bifidobacterium*, and *Streptococcus thermophilus*	Improvement of lipid profile and inflammatory markers
*Bacillus coagulans*, *Lactobacillus rhamnosus*, and *Lactobacillus helveticus*	Improvement of the inflammatory profile due to limited CRP production
*Lactobacillus paracasei*	Improvement of lipid profile
*Akkermansia muciniphila*	Increased insulin sensitivity, reduced fasting insulin, and improvement of lipid profile
*Faecalibacterium*	Improvement of kidney function due to reduced urea, IL-6, and other inflammatory and oxidative stress markers
*Bifidobacterium longum*	Improvement of kidney function due to reduced uremic toxins
*Lactobacillus* acidophilus	Improvement of kidney function due to reduced uremic toxins
*Bifidobacterium animalis* subsp. lactis	Modulation of 5-hydroxytryptamine levels
*Bifidobacterium bifidum*	Neuroprotection

*IL-6*, Interleukin 6; *SCFAs*, short-chain fatty acids; *TMAO*, trimethylamine N-oxide; *CRP*, C reactive protein

Once larger studies are available, clinicians could use many clinical implementation strategies to educate patients about probiotic medications. Firstly, it is worth noting that choosing the strain-specific supplement for each condition is of utmost importance (e.g., PS128 for mood and *Akkermansia* for metabolic health). Avoiding general “probiotic” labels by checking the strain, dosage, and delivery form will also be a valuable strategy for personalized treatment strategies for patients at higher risk for cardiovascular, brain, and kidney diseases. Stimulating the interprofessional team’s use of probiotics will also be a valuable strategy for this intervention. Training nurses, dietitians, and pharmacists to screen for gut-related symptoms and make initial probiotic recommendations would be of great value for improving the medication acceptance by patients and medical centers, but also help improve patients’ quality of life and treatment efficacy with natural strategies that limit adverse effects. In this scenario, patient education might be helpful. Clinicians might provide handouts explaining how probiotics work, what outcomes they are expected to offer and when, and the importance of a consistent diet and medication regimen. While describing how probiotics work, clinicians might be able to advance their knowledge of how microbiota influences inflammation through the gut-brain-kidney-heart axis. While explaining the treatment duration and expected symptom improvement, clinicians must highlight the chronic use of probiotics as a necessary strategy. Lastly, when combined with lifestyle changes, probiotics may advance even more health strategies. These include, for example, a great fiber intake, which enhances probiotic effects.

## Conclusion

Our findings show that probiotics cause several benefits in the intestine-brain–heart-kidney axis. Among the main benefits for heart and cardiovascular diseases are the reduction of TMAO, TNF-α, IL-6, and serum lipid levels. Therefore, there is a reduction in the likelihood of developing diseases such as atherosclerosis and heart failure. Furthermore, probiotic supplements that are beneficial for the proper functioning of the intestine increase the variability of species advantageous to the intestine and promote better intestinal motility and a reduction in abdominal injuries. In addition, kidneys also benefit from these supplements, as they reduce sodium levels and uremic toxins. In the brain, these microorganisms cause numerous improvements, as they help reduce symptoms of depression and anxiety, improve sleep quality, and suppress the progression of brain atrophy.

Evidence suggests that probiotic efficacy highly depends on host-specific factors, including baseline microbiome composition, genetics, diet, and immune response statuses. Due to these reasons, personalized treatment approaches to tailor the best probiotic therapy to individual microbiome profiles are of utmost importance. Prior microbiome profiling could help identify responders and non-responders, enabling precision-targeted probiotic strategies to yield more positive outcomes for clinically susceptible patients. In this case, future research must prioritize stratifying the included patients by microbiome data, bacterial metabolic signatures, or immune phenotyping to predict individual responses to specific probiotic strains better. In addition, engineered strains, which are the next generation of probiotics made with artificial intelligence or machine learning strategies with defined consortia and functions of beneficial microbes, should be incorporated since they have the prospect of being disease- and patient-specific. By embracing personalized clinical strategies, probiotics can be better positioned regarding wellness supplementation to achieve precision therapeutic outcomes.

## Challenges and Future Perspectives

As mentioned, probiotics are related to several benefits in the intestine-brain–heart-kidney axis. However, there are still many challenges that must be taken into consideration. Perhaps the first challenge is that the systemic effects of probiotics have not yet been fully established. New research is needed to demonstrate the molecular impact of these microorganisms, unraveling their effects on mechanisms of action and in the inflammatory and pro-oxidative cascades. Regulatory measures are also needed regarding the type of probiotic to be used and the effective and safe dosages to obtain the desired effects. Individual variability concerning probiotics must also be considered. In this sense, personalized medicine significantly contributes to getting the best preventive and therapeutic impact.

In the included studies, probiotic dosages varied significantly, from 1 × 10^9^ CFU twice daily (for *L. plantarum*) to over 900 billion CFU/day (for Vivomixx®), highlighting formulation challenges, treatment durations, and delivery methods. However, the relationship between dose and therapeutic benefit is still unclear since small doses can have better efficacy depending on the utilized strain for specific health problems. The treatment periods and durations also varied greatly, from 7 days (in the AKI study) to up to 6 months (for hemodialysis and cardiovascular trials). This suggests that condition-specific durations, despite probiotic strain, may also be a reality. However, standard guidance is also lacking. The delivery methods utilized varied significantly between the included studies. However, capsules were the most common method (e.g., Simbin-RNL®, Bio-Kult, NVP-1704), offering controlled dosing but requiring gut viability for optimal outcomes. In some studies, fermented dairy was also utilized as a delivery method for probiotic interventions, including unfermented milk with *B. longum*. Sachets have also provided alternative vehicles. However, this might induce bioavailability alterations due to packaging techniques involving extending shelf-life. Prebiotics and symbiotics can also synergize with probiotics to improve efficacy and health outcomes, promoting more nuanced and prominent colonization. However, these interactions were less frequently observed during the evaluation of the included studies. 

## Data Availability

No datasets were generated or analysed during the current study.
